# Human iPSC-derived neural stem cells with *ALDH5A1* mutation as a model of succinic semialdehyde dehydrogenase deficiency

**DOI:** 10.1186/s12868-022-00755-3

**Published:** 2022-12-16

**Authors:** Xiaodan Chen, Minzhi Peng, Yanna Cai, Chengcheng Zhou, Li Liu

**Affiliations:** Department of Genetics and Endocrinology, Guangzhou Women and Children’s Medical Center, Guangzhou Medical University, Guangzhou, People’s Republic of China

**Keywords:** SSADH-D, Human induced pluripotent stem cells, CRISPR/Cas9, Neural stem cells

## Abstract

**Background:**

Succinic semialdehyde dehydrogenase deficiency (SSADH-D) is an autosomal recessive gamma-aminobutyric acid (GABA) metabolism disorder that can arise due to *ALDH5A1* mutations, resulting in severe, progressive, untreatable neurodegeneration. SSADH-D is primarily studied using simplified models, such as HEK293 cells overexpressing genes of interest, but such overexpression can result in protein aggregation or pathway saturation that may not be representative of actual underlying disease phenotypes.

**Methods:**

We used a CRISPR/Cas9 approach to generate human iPSC cell lines bearing *ALDH5A1* mutations. Through screening, two different mutant cell lines, NM_001080.3: c.727_735del (p.L243_S245del) and NM_001080.3: c.730_738del (p.A244_Q246del), were obtained. We induced iPSCs to neural stem cells and analyzed the characteristics of *ALDH5A1* mutations in stem cells.

**Results:**

The human iPSC and NSC cell lines presented typical stem cell–like morphology. We found changes in *ALDH5A1* expression and GABA accumulation in the different cell lines. In addition, by analyzing the cDNA between the wild-type and the mutant cell lines, we found that the mutant cell lines had a splicing variant.

**Conclusions:**

iPSCs represent a promising in vitro model for SSADH-D that can be used to study early central nervous system developmental alterations and pathogenic mechanisms.

**Supplementary Information:**

The online version contains supplementary material available at 10.1186/s12868-022-00755-3.

## Introduction

*ALDH5A1* encodes the SSADH protein that is responsible for a step in the breakdown of gamma-aminobutyric acid (GABA), an inhibitory neurotransmitter present in neural cells. *ALDH5A1* mutations lead to a rare inborn error in the metabolism of GABA. As a consequence, physiologic fluids from individuals harboring *ALDH5A1* mutations accumulate gamma-hydroxybutyrate (GHB), and succinic semialdehyde dehydrogenase deficiency (SSADH-D) occurs. Patients with SSADH-D present with late-infantile to early-childhood onset of slowly progressive neurodegeneration or static encephalopathy [1, 2]. While many researchers have sought to understand this condition, knowledge about many aspects of the pathophysiology of SSADH-D remains insufficient because of the difficulty of establishing a nervous system model [3]. SSADH-D has been studied using simplified models, such as HEK293 cells overexpressing genes of interest. However, overexpression of proteins can result in protein aggregation or pathway saturation that may not represent actual underlying disease phenotypes [3]. Furthermore, dermal fibroblasts of SSADH-D patients have a short life span and low expression levels of SSADH, limiting their application for study. Several animal models of SSADH-D have been reported [4–7], however, making a mouse model is much more expensive and time consuming than obtaining a cell model. Induced pluripotent stem cells (iPSCs) provide a well-defined source of tissue-specific cells and are invaluable disease modeling tools. Other advantages of iPSCs include their unlimited proliferation and multi-directional differentiation.

In this study, we generated iPSCs with *ALDH5A1* mutation as a disease research model of SSADH. NM_001080.3: c.727_735del is a compound heterozygous mutation in *ALDH5A1* that we previously reported [8]. We used the CRISPR/Cas9-mediated editing system to generate mutated hiPSC lines harboring this mutation. A single guide RNAs (sgRNA) targeting *ALDH5A1* NM_001080.3: c.727_735del and the ribonucleoprotein Cas9 were used to edit the gene via homologous recombination. The sgRNA was engineered with an endogenous fluorescent reporter-enhanced green fluorescent protein (sgRNA-eGFP). The edited knock-in cell lines expressed the fluorescent reporter that was detectable by flow cytometry and confirmed transfection. Through screening, two different mutant cell lines, NM_001080.3: c.727_735del (p.L243_S245del) and NM_001080.3: c.730_738del (p.A244_Q246del), were obtained. These two cell lines and the control iPSCs were further differentiated into neural stem cells (NSCs). *ALDH5A1*-mutated cell lines were then used for disease modelling, with the wild-type as the control line. Our results indicated that *ALDH5A1* expression significantly increased when iPSCs were induced into NSCs. The absolute amount of GABA in NSCs was less than that in iPSCs. The accumulation rate of GABA in the mutant NSCs was higher than that in the mutant iPSCs. Interestingly, the two cell lines with only one amino acid mutation difference exhibited various characteristic changes when iPSCs were induced to NSCs. This finding suggested that the constructed SSADH disease models in iPSCs will be beneficial for studies on disease mechanisms.

## Results

### Generation of knock-in hiPSC lines with CRISPR/Cas9 technology

**Fig. 1:**
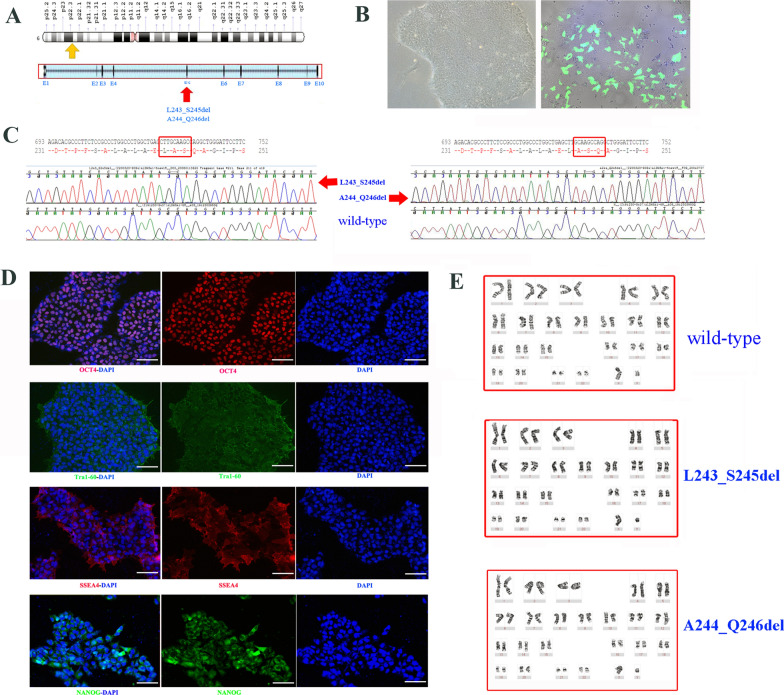
Characterization of *ALDH5A1*-mutated iPSC lines (p.L243_S245del and p.A244_Q246del). **A** Illustration showing the CRISPR/Cas9 strategy for the *ALDH5A1* gene. (The yellow arrow marks the position of the gene on the chromosome); The sgRNA targets the two mutations in exon 5 indicated by the red arrow. **B** Images showing typical stem cell morphology and efficiency of transfection (magnification, × 10). **C** Sanger Sequencing and alignment of sequencing results showing deletions in the *ALDH5A1* gene (p.L243_S245del mutant, left; p.A244_Q246del mutant, right). **D** Representative images of immunofluorescence staining of wild-type cells for pluripotency markers OCT4, Tra1-60, SSEA-4 and NANOG; DAPI was used to stain for nuclei. Scale bar = 100 μm. (iPSC, the wildtype cell lines) **E** Results of the karyotype analysis for the three cell lines

We used a CRISPR/Cas9 editing strategy to engineer a hiPSC line with mutations in the *ALDH5A1* locus (Fig. 1A). First, two different sgRNAs were designed in silico using the online CRISPR Design Tool (http://crispr.mit.edu/) [9]. The efficiency of each candidate was tested in vitro using hiPSCs. We evaluated the transfection efficiency by assessing eGFP fluorescence and estimated the editing efficiency via sequencing to single out two sgRNAs. The Cas9 plasmid contained a nonviral vector to express Cas9 protein and an EF1A core promoter sequence flanked by T2A sites. The sgRNA plasmid contained a nonviral vector of gRNA for mammalian cells and the eGFP coding gene with the U6 core promoter sequence flanked by P2A sites.

hiPSCs were transfected with the sgRNA plasmid and an expression vector carrying Cas9 (Fig. 1B) The successfully transfected cells were screened with flow cytometry; the results revealed that secondary sorting improved the acquisition of positive-edited cells. Single hiPSC clones were obtained and amplified to obtain DNA for sequencing (Fig. 1C) and find homozygous mutated hiPSC clones. Whole-genome sequencing confirmed no other significant changes in the mutant cell lines. The target fragment of the *ALDH5A1* gene was edited correctly.

The iPSC cell line presented a normal karyotype (Fig. 1E) and was free of mycoplasma. The mutated hiPSC was also confirmed to have a normal karyotype.

### Immunofluorescence of iPSCs

The cell line presented typical stem cell-like morphology (data not shown). Immunofluorescence assays showed the expression of the pluripotency markers, such as OCT4, SSEA-4, TRA-1–60, and NANOG (Fig. 1D) in the parental and edited hiPSC lines.

**Fig. 2:**
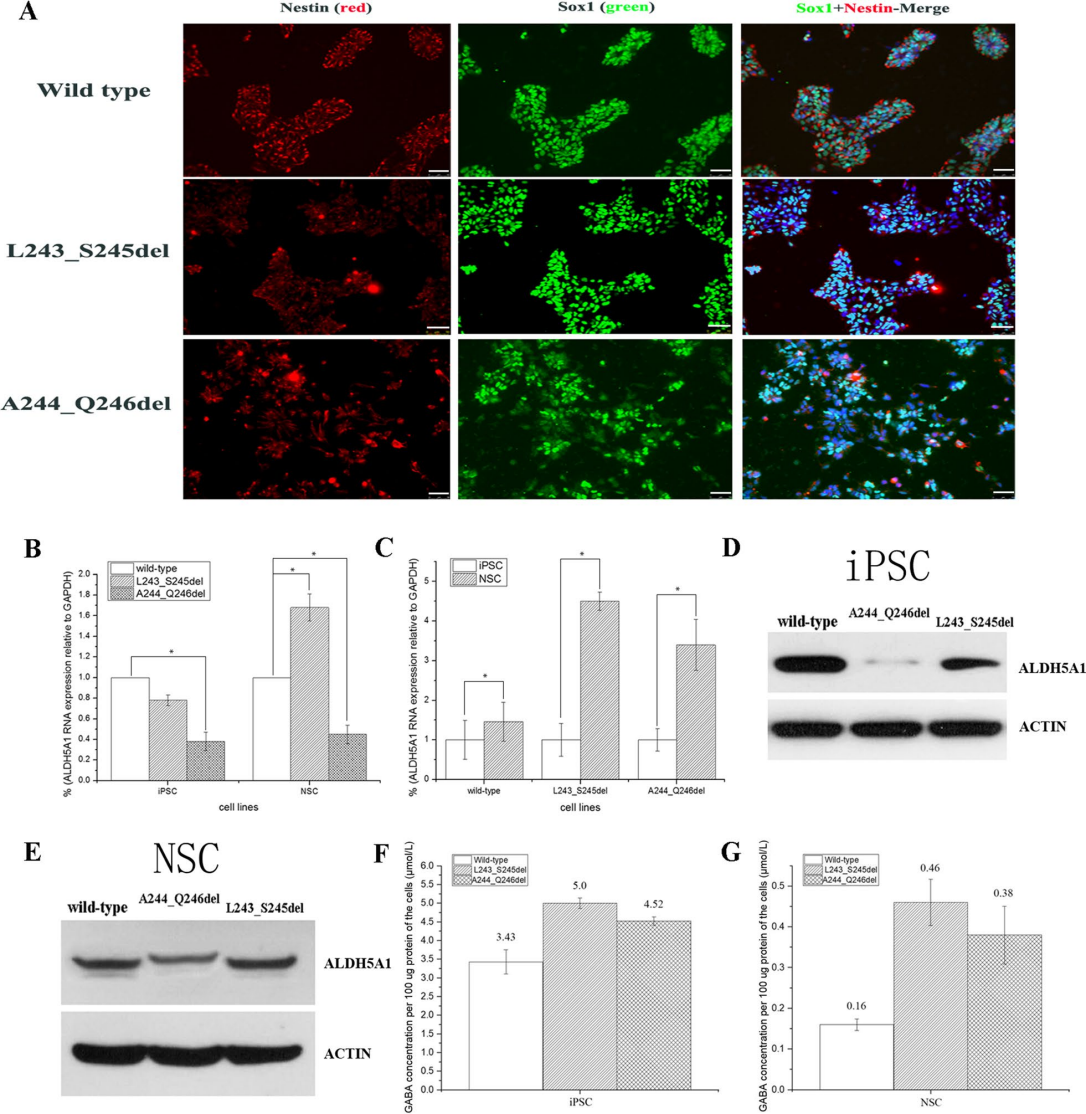
Characterization of *ALDH5A1*-mutated NSC lines (p.L243_S245del and p.A244_Q246del). **A** Immunofluorescence staining of iPSCs induced to neural stem cell lines (NSCs): Sox1 [scale bar = 50 μm, (white)], Nestin [scale bar = 50 μm, (white)]. **B** *ALDH5A1* gene expression was measured by real-time quantitative PCR analysis and normalized to GAPDH. mRNA expression in iPSC lines and induced NSCs. The histogram shows the average value calculated by three pairs of qPCR primers mentioned in Table 1. The p.A244_Q246del mutant cells exhibited significantly reduced gene expression of *ALDH5A1* compared with wild-type cells (*P < 0.01). However, the gene expression of *ALDH5A1* of p.L243_S245del mutant showed a slight decrease, and it increased significantly and was higher than that of wild-type when induced to NSCs (*P < 0.01). (Each experiment was repeated three times, data are presented as mean) **C** The expression of the *ALDH5A1* gene was increased significantly when human pluripotent stem cells were induced into neural stem cells (*P < 0.01). **D**–**E** Western blot analyses revealed that *ALDH5A1* in the two iPSC mutant cell lines decreased. Each experiment was repeated three times. Ratio of the gray value of the target protein to the internal reference (from left to right) is 0.97, 0.06, 0.47 (iPSC); 0.43, 0.29, 0.46 (NSC). (see Additional files 1, 2) **F** Analysis of GABA accumulation in iPSC cell lines by denaturing high-performance liquid chromatography-tandem mass spectrometry. **G** Analysis of GABA accumulation in NSC cell lines by denaturing high-performance liquid chromatography-tandem mass spectrometry. In 2B, 2C, 2F, and 2G, data are shown as mean ± standard deviation

Sox1 and Nestin were expressed in wild-type and mutant NSC lines (Fig. 2A).

### mRNA and protein expression levels in iPSC and NSC lines

We next evaluated *ALDH5A1* mRNA levels in the iPSC and NSC parental and mutated cell lines (Fig. 2B). The results showed that p.A244_Q246del cells may be more unstable than p.L243_ S245del cells because of the significant difference in *ALDH5A1* mRNA between the wild-type and p.A244_ Q246del cells (Fig. 2B); the same result was observed in western blot analyses of iPSC cell lines (Fig. 2D). However, there was only a slight decrease in gene and protein expression of *ALDH5A1* in p.L243_ S245del compared with wild-type. And the gene expression increased significantly than that of wild-type when induced to NSCs (Fig. 2C). Interestingly, the expression of *ALDH5A1* in NSCs with the same mutation type was also significantly higher than that in iPSCs because of the tissue-specific expression (Fig. 2C).

### GABA accumulation in iPSCs and NSCs

We evaluated intracellular GABA accumulation using HPLC (Fig. 2F and G), and the results showed that the GABA accumulation in the two mutant cell lines was higher than that of the wild-type in iPSCs and NSCs. The GABA content in the two iPSC mutant lines was 50% higher than that in wild-type, and levels in NSCs were three-fold higher than the wild-type. Our results showed that differentiation of iPSCs into NSCs led to increased *ALDH5A1* expression (Fig. 2C), which may be why the GABA concentration detected in NSCs was generally lower than that of the iPSCs. The p.L243_S245del mutant line had similar (iPSCs) or higher (NSCs) *ALDH5A1* RNA expression than wild-type line (Fig. 2B) and similar protein expression (Fig. 2D and E), yet the GABA concentration was higher in both iPSCs and NSCs, indicating that the p.L243_S245del mutant *ALDH5A1* protein is less active than the wild-type protein. The p.A244_Q246del iPSCs express significantly less *ALDH5A1* protein (Fig. 2D), which explains why the GABA concentration is higher compared with levels in the wild-type. However, the GABA concentration in the p.A244_Q246del line was higher relative to the wild-type line even after differentiation into NSCs, where the protein levels of *ALDH5A1* were similar (Fig. 2E). This further suggests that the p.A244_Q246del mutant protein may be less active than the wild-type protein.

### mRNA sequence analysis after genome editing in iPSC

**Fig. 3:**
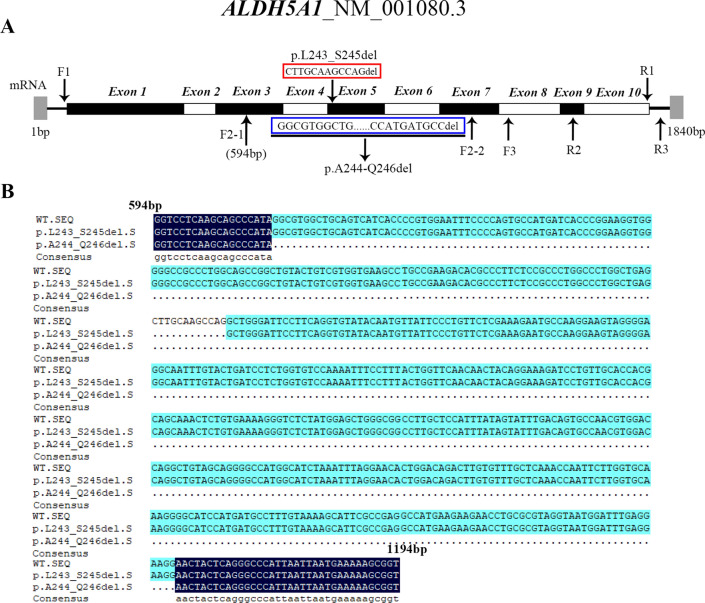
The cDNA sequencing of *ALDH5A1*-mutated iPSC lines (p.L243_S245del and p.A244_Q246del) and the wild-type. **A** The letters F1, R1, F2-1, F2-2, R2, F3, and R3 indicate the location of each primer for cDNA sequencing. **B** Comparison of the cDNA sequencing results among two mutant cell lines and the wild type. The mRNA splicing variant detected in L243_S245del results in the deletion of 12 bases in exon 5 and the mRNA splicing variant detected in p.A244_Q246del mutant lacks 545 bases encompassing exons 4 to 6 and parts of exons 3 and 7

Finally, we performed mRNA sequence analysis. In the first round of amplification, we used F1 and R1 primers to amplify the entire mRNA, and the fragment size was 1637 bp. F2-1 sequencing of the A244_Q246del sample was successful, and F2-2 sequencing failed. The F2-1 sequencing result is the range of F2-2 sequencing. There may be splicing variants between the sequences between F2-1 and F2-2. Through analyses of the sequencing results, we found that the A244_Q246del has a large fragment splicing variant, and L243_S245del has 12 base splicing variants (Fig. 3, Table 1).

**Table 1:** Reagents and primers details

Antibodies used for immunocytochemistry Antibody	Dilution	Cat # and RRID
Pluripotency markers		
Mouse anti-OCT4	1:200	CST Cat.75463
Rabbit anti-NANOG	1:200	CST Cat.4903 T
Mouse anti-TRA1-60	1:500	Abcam Cat.ab/6288
Mouse anti-SSEA4	1:500	CST Cat.MC813
NSC differentiation markers		
Mouse anti-Nestin	1:200	ABcam.Cat.ab18102
Rabbit anti-sox1	1:200	CST Cat.4194 s
Secondary antibodies		
Mouse anti-IgG	1:1000	CST Cat.4409
Goat-anti-Rabbit	1:500	Servicebio. Cat.GB25303

Primers	Target	Forward/Reverse primer (5′-3′)	
Targeted mutation	ALDH5A1-Exon5	ACGTGACTTTAGCACTAATAAGA	AGAGCTTTTAACACTCTGCTGGA
RT-qPCR Primers	ALDH5A1-RNA-1 (exons 8–10)	GGCACCAGTTATCAAGTTCG	ACTCCACCAAAAGGGCACTC
	ALDH5A1-RNA-2 (exons 3–4)	AGTCATCACCCCGTGGAATTT	GAGAAGGGCGTGTCTTCGG
	ALDH5A1-RNA-3 (exons 5–6)	AGGGGAGGCAATTTGTACTGA	GTGGTGCAACAGGATCTTTCC
	GAPDH	GCACCGTCAAGGCTGAGAAC	TGGTGAAGACGCCAGTGG
	β-ACTIN	CATGTACGTTGCTATCCAGGC	CTCCTTAATGTCACGCACGAT
cDNA sequencing	F1/R1	TTCCTGTCGCCGTCGTTGC	ATCCTACAAGCCCCCGTAACAC
	F2-1	GGTCCTCAAGCAGCCCATA	
	F2-2	TTTGTAAAAGCATTCGCCG	
	F3	GATGCCGTTTCTAAAGGTGC	
	R2		AAAATAACCTGCTAACCCAACATC
	R3		TTACAAGGACTGGATGAGTTCT

## Discussion

The biochemical hallmark of SSADH-D is an increase in the concentration of GABA and GHB in body fluids, such as blood, urine, and cerebrospinal fluid. These two substances mainly affect the nervous system. Thus, SSADH-D is often characterized by nervous system lesions [10, 11]. SSADH-D has been studied using simplified model symptoms such as HEK293 cells overexpressing genes of interest, but such overexpression can result in protein aggregation or pathway saturation that may not represent actual underlying disease phenotypes [3]. The short life span of dermal fibroblasts and low expression levels of SSADH in these cells further limit the application of dermal fibroblasts in a patient. Thus, more in vitro approaches with cellular models should be developed. For instance, iPSC models of various diseases have emerged [12–16]. In the present study, we successfully generated two SSADH-D iPSC lines via CRISPR/Cas9 genome engineering combined with flow cytometry and a clonal loop. The advantage of this model was that iPSCs can be differentiated into all cell types of the body, especially those representing the target tissue cells, such as NSCs. Through the induction of iPSCs, NSCs harboring wild type and mutant *ALDH5A1* showed the characteristic changes of SSADH-D. Comparing the expression of *ALDH5A1* and the amount of GABA between iPSCs and NSCs shows an interesting finding (Fig. 2F and G). When iPSCs were induced into NSCs, *ALDH5A1* expression increased in the wild type and mutant cells, but the degree of increase differed, and the increase in mutant cell lines was more apparent (Fig. 2C). The GABA accumulation in mutants in NSCs was more pronounced than that of the mutants in iPSC lines (Fig. 2F and G). However, in comparison with NSCs, iPSC likely tolerated higher GABA concentrations. This observation emphasizes that more SSADH was needed to help metabolize GABA in NSCs. GABA accumulation could not be reduced even when the gene expression increased because of *ALDH5A1* mutation.

Furthermore, the generation of L243_S245del and A244_Q246del allowed us to conclude about the in vitro function of the splicing region between exons 4 and 5 in *ALDH5A1*. As shown in Fig. 2B, the expression of the A244_Q246del mutant was only 30%–40% of that of the wild-type before and after iPSCs induced to NSCs. However, the decrease in the *ALDH5A1* expression in the L243_S245del mutant (75% of wild-type) in iPSC lines was not statistically significant compared with that in the wild-type. Even after induction, *ALDH5A1* expression was higher in the L243_S245del mutant than the wild-type (Fig. 2C). These results showed that although only one amino acid difference existed between these two mutations, the two mutant cell lines harbored different mechanisms affecting protein expression. This hypothesis was tested by sequencing the cDNA of the target gene in the two different mutants. As expected, the A244_Q246del mutant (mutation in exon 5) severely affected RNA splicing, resulting in a 545 bp deletion encompassing exons 4 to 6 and parts of exons 3 and 7 of *ALDH5A1* (Fig. 3B). PCR amplification of cDNA obtained from the p.A244_Q246del line using primers F1 and R1 yielded two bands with similar intensities, suggesting similar abundance of the full-length and spliced product (data not shown). L243_S245del only affected the deletion of 12 bases (CTTGCAAGCCAG) in exon 5 (Fig. 3B). Thus, this observation was the fundamental reason for the different changes in *ALDH5A1* expression before and after iPSC induction between the two mutant cell lines. Akaboshi et al. [17] concluded that the mutations in a short stretch between aa 223 and 268 (encoded by exons 4 and 5) are not entirely random, and this region in the gene may be vital. This finding was helpful for further studying the functional contribution of the vital region of the *ALDH5A1* gene. Therefore, the iPSC disease model may be useful for research on SSADH-D mechanisms.

A correct diagnosis is the first step in treating rare diseases, and it is the basis of disease mechanism research. For rare diseases, the availability of validated samples from patients with a specific disease is usually low, limiting the possibilities of using these samples. iPSC disease models can be differentiated into various target cells because of the pluripotency of iPSCs. In our study, the iPSC disease model was successfully differentiated into NSCs. This model was utilized to detect changes in *ALDH5A1* expression and GABA accumulation in two mutant cell lines. This may be a good cell model for drug screening because the therapy-induced reduction of GABA in the periphery may be a vital issue for the development of future therapies for SSADH-D [18].

In conclusion, CRISPR-based genome editing of iPSCs shows potential for future studies on the pathogenicity of diseases. Our research demonstrated that iPSCs could be helpful for SSADH-D disease modelling.

## Materials and methods

### Cell line

The hiPSC line (DYR0100), derived from a human prepuce cell of a healthy boy donor, and was procured from Hunan Fenghui Biotechnology Company (ATCC-ACS-1011TM).

### Gene editing

#### sgRNA design and testing

sgRNAs were designed in silico using the CRISPR Design Tool (http://crispr.mit.edu/). The sgRNAs were designed to target the NM_001080.3: c.727_735del region of *ALDH5A1*. Two sgRNAs with a high fraction of efficacy were selected. The two oligonucleotides for sgRNAs were cloned into two VB UltraStable competent cells. The sgRNA1 sequence was: 5′-TATAGCTTGCAAGCCAGGCT-3′, and the protospacer adjacent motif (PAM) sequence was GGG. The sgRNA2 sequence was: 5′-TTATAGCTTGCAAGCCAGGC-3′, and the PAM sequence was TGG.

hiPSC cells (300,000/well) were plated in a 6-well culture plate. On the following day, the cells were transfected with 2 µg sgRNA1 and Cas9-carrying plasmid using DNA Lipofectamine™ Stem transfection reagent (ThermoFisher, Cat# STEM00003). Cells were cultured for 24 h and then screened by flow cytometry (BD FACS Aria SORP, USA). sgRNA1 was more efficient than sgRNA2, and they had editorial effects.

#### ssDNA for homologous directed repair

Single-stranded donor oligonucleotides were synthesized by Vector Builder and provided at 10 µM concentrations. The sequence was TTTTTTTTTTTTTTCAGTTTGGTAAATTTTGGCAAGTTTGCTTTTCTCTTTATAGCAGGCTGG GATTCCTTCAGGTGTATACAATGTTTTCCCTGTTCTCGAAAGAATGCCAAGGA. At the time of transfection, 2 µl was added to each well of the 6-well plate.

### Cell culture

#### hiPSCs culture and transfection

hiPSCs were cultured in a PGM1 medium (Cellapy, CAT# CA3001500) with 0.5% Plasmocin prophylactic on matrix-coated (Corning Matrigel hESC-Qualified) plate and maintained at 37 °C in humidified air with 5% CO_2_. Cells were passaged with Cellapy Cell Dissociation Reagent (Stem cell, CAT# 07174) every 3–4 days and plated at a density of 2 × 10^4^ cells/cm^2^ with a split ratio of around 1:6. During cell generation, 10 µM Y-27632 (STEMCELL Technologies, CAT# 72,302) was added.

Cells were transfected with a complex formed with the Cas9 plasmid (VB190801_1165nbd) and two sgRNA plasmids (VB190801-1166xkk and VB190801-1168fdu), constructed by VectorBuilder, using Lipofectamine Stem transfection reagent (Invitrogen, CAT #STEM00001).

The detailed steps were as follows:

**Table:** 

Steps	Tube	Component	Per well of a 6-well plate
1	1	Opti-MEM™ I Medium	100 µl
		Lipofectamine™ Stem Reagent	4 µl
2	2	Opti-MEM™ I Medium	100 µl
		DNA	2 µg*
3	Add diluted DNA to diluted Lipofectamine Stem Reagent		
4	Incubate for 10 min at room temperature		
5	Add DNA-lipid complex to cells (200 µl/per well)		
6	Incubate and monitor the transfected stem cells at 37 °C for 2 days		

*Equimolar amounts of Cas9 plasmid DNA and gRNA plasmid DNA were added

### Screening of positive clones with targeted homozygosity

After 48 h of transfection, the cells were harvested with StemPro Accutase (Stem Cell, CAT. #At-104) and sorted on a BD FACS Aria SORP (BD Biosciences). eGFP-positive cells were collected, re-sorted to remove false-positive cells, and cultured in 12-well plates. For homologous directed repair screening, 1 week after cultivation, a portion of the cells of each colony was removed and DNA was extracted using the DNeasy Blood and Tissue Kit (Qiagen) for PCR amplification and sequencing. Sequencing results confirmed successful editing and cells were selected in a 10-cm dish by clone ring (Sigma, Aldrich, CAT# C7983-50EA).

### Chromosome analysis and whole-genome sequencing for iPSC

Cultured cells were incubated in 50 ng/ml colcemide solution (Gibco, CAT# 15210–040) for 1 h, subjected to hypotonic treatment in 0.075 M KCl for 20 min at 37 °C, fixed with Carnoy solution (3:1 v/v methanol/acetic acid) twice for 20 min each and spread on a wet cooled microscopic slide with a plastic transfer pipette to obtain chromosome preparations for karyotype analysis. After the specimen was air-dried, the slide was incubated on a heating plate at 82 °C for 2.5 h. After 10 min of Giemsa staining, changes in karyotype were observed under a phase-contrast microscope.

The two successfully edited homozygous cell lines and the unedited cell lines were sent to BGI for genome-wide sequencing to confirm that no undesired editing was present in the cell lines.

### Immunofluorescence staining

The cells were fixed in 4% PFA for 20 min and washed three times with phosphate-buffered saline (PBS). Cells were permeabilized with 1 ml of 0.5% Triton-X 100 for 20 min and blocked with 5% BSA for 1 h at room temperature. Cells were incubated with primary antibodies (against OCT4, TRA-1–60, SSEA, and NANOG) at 4 °C for overnight and then washed three times with PBS. Cells were then incubated with secondary antibodies in the dark at room temperature for 2 h. Nuclei were stained with 0.5 µg/ml DAPI, and images were acquired with a ZOE fluorescence cell imager (Bio-Rad).

### Induction and identification of NSCs

iPSC Neural Induction Medium (Gibco, CAT# A1647801) was used to differentiate human iPSCs into NSCs following the manufacturer’s instructions. The staining was performed to identify NSCs as in Section above. Primary antibodies were Sox1 and Nestin antibodies.

### Real-time PCR analysis

Total mRNA was extracted from cells by Trizol (Invitrogen). First-strand cDNA synthesis was performed on 1 µg of the total RNA. Real-time PCR for *ALDH5A1, GAPDH* and *β-ACTIN* was performed using ChamQ™ Universal SYBR-qPCR Master Mix (version7.1, Vazyme); reactions were performed in triplicate. The following primers were used for gene detection: *ALDH5A1*-1, forward 5′ -GGC ACC AGT TAT CAA GTT CG-3′ and reverse 5′ -ACT CCA AAA GGG CAC TC-3′; *ALDH5A1*-2, forward 5′ -AGT CAT CAC CCC GTG GAA TTT-3′ and reverse 5′-GAG AAG GGC GTG TCT TCG G-3′; *ALDH5A1*-3, forward 5′-AGG GGA GGC AAT TTG TAC TGA-3′ and reverse 5′-GTG GTG CAA CAG GAT CTT TCC-3′; *GAPDH*, forward 5′-GCACCGTCA AGG CTG AGA AC-3′ and reverse 5′-TGG TGA AGA CGC CAGTGG A-3′; and *β-ACTIN*, forward 5′–3′ CATGTACGTTGCTATCCAGGC and reverse 5′–3′ CTCCTTAATGTCACGCACGAT (Table 1). The target gene (*ALDH5A1)* expression levels were calculated using the comparative threshold cycle (Ct) method with the following formula: ΔCt = Ct (gene of interest) − Ct (*GAPDH* and *β-ACTIN*); the 2^−ΔΔCt^ was calculated with the following formula: ΔΔCt = ΔCt (control group) − ΔCt (experimental group) to determine the relative expression. In each experiment, each sample was analyzed in triplicate. The result of *β-ACTIN* is not show in the figure.

### Western blot

Cells were lysed with RIPA lysis buffer (Beyotime, Beijing, China) containing 1% PMSF (Beyotime, Beijing, China). Protein (15 μg) was loaded on 10% SDS-PAGE gels for electrophoresis and transferred to PVDF membranes. The membranes were blocked with 5% non-fat dry milk in TBS containing 0.1% Tween 20 for 1.5 h at room temperature and then incubated with antibody for SSADH (AffinitY, Cat# DF12820) and *β*-actin (Multi Sciences, Hangzhou, China) at 4 °C overnight. The membranes were then incubated with HRP-conjugated secondary antibody (Multi Sciences, Hangzhou, China) for 2 h at room temperature. Signals were detected using film by darkroom exposure (Servicebio, G2019, China). In each experiment, each sample run on the SDS-PAGE gel in duplicate.

### High-performance liquid chromatography–tandem mass spectrometry (HPLC–MS/MS)

GABA in iPSC and NSC cell lines was determined with HPLC–MS/MS, as previously described [19]. The samples used were total protein obtained by two duplicate experiments (n = 2).

### cDNA sequencing

RNA from wild-type and mutant cell lines was extracted and reverse transcribed to cDNA. cDNA was amplified using the following primers: F1: TTCCTGTCGCCGTCGTTGC, R1: ATCCTACAAGCCCCCGTAACAC. For segmented sequencing analysis, we used the following primers: F2-1: GGTCCTCAAGCAGCCCATA, F2-2: TTTGTAAAAGCATTCGCCG, R2: AAAATAACCTGCTAACCCAACATC; F3: GATGCCGTTTCTAAAGGTGC, and R3: TTACAAGGACTGGATGAGTTCTG. The locations of the primers are shown in Fig. 3. (Table 1).

## Supplementary Information

**Additional file 1:** full-length blot gels are presented in Supplementary Figure 2D.

**Additional file 2:** full-length blot gels are presented in Supplementary Figure 2E.

## Data Availability

The datasets analyzed during the current study are available in the Clinvar repository, [https://www.ncbi.nlm.nih.gov/clinvar, Submission ID: SUB11566734].
