# Supplementary figures and images for: Human iPSC-derived neural stem cells with ALDH5A1 mutation as a model of succinic semialdehyde dehydrogenase deficiency
Source: BMC Neurosci. 2022 Dec 16;23:77. doi: 10.1186/s12868-022-00755-3 (PMC9756581; doi:10.1186/s12868-022-00755-3)

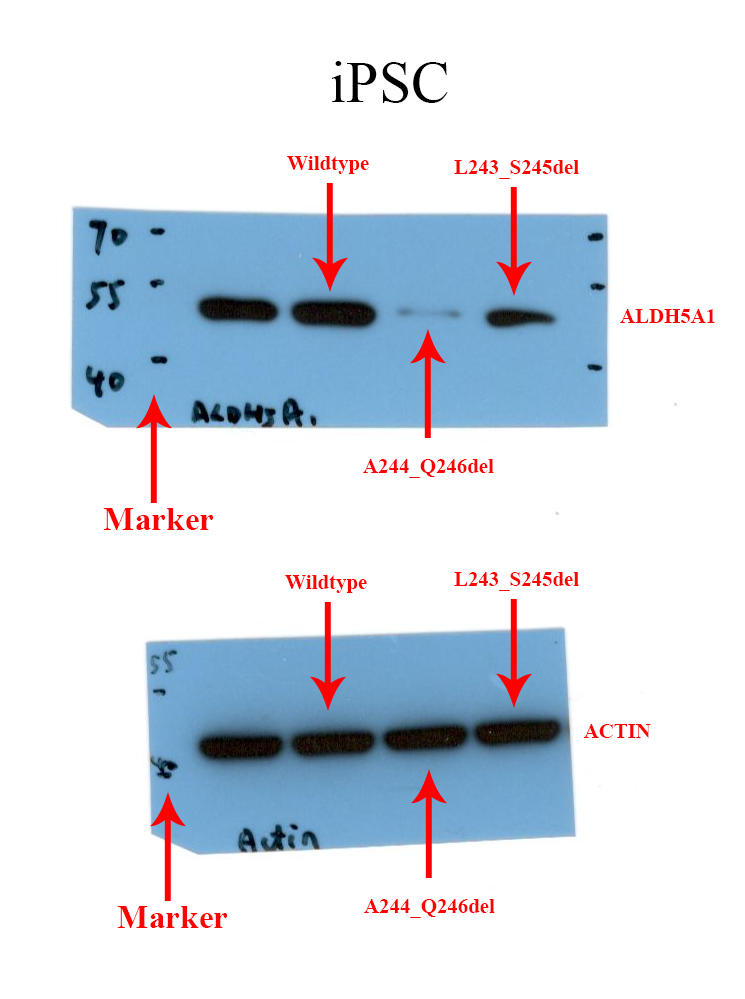

Supplement: Supplementary file 1 — Additional file 1: full-length blot gels are presented in Supplementary Figure 2D. [file 12868_2022_755_MOESM1_ESM.tif]

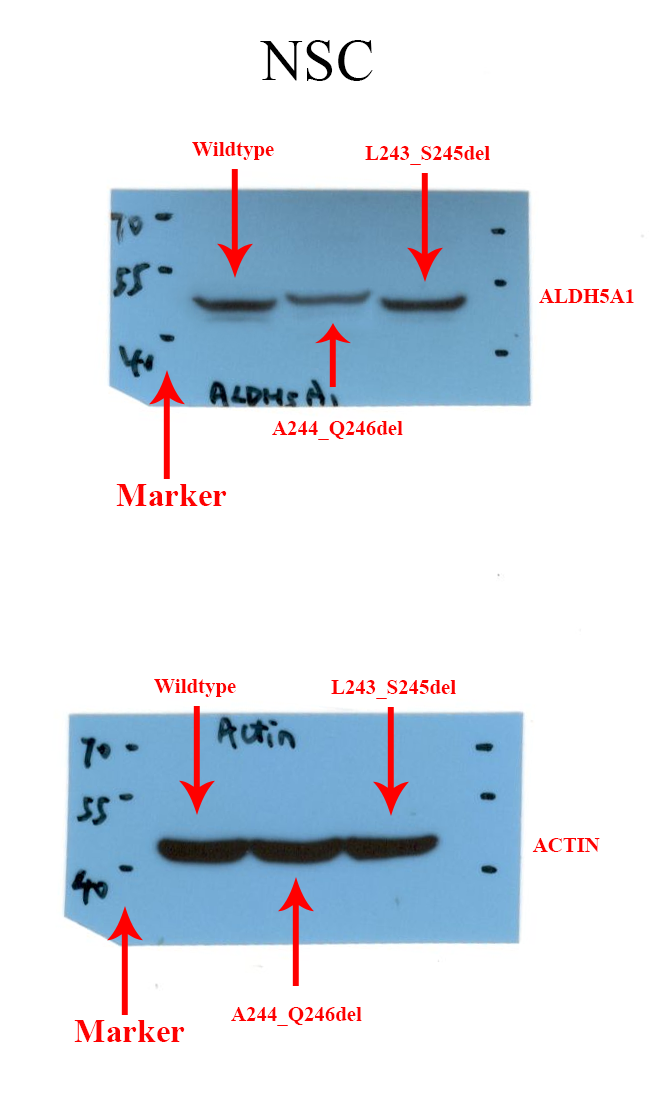

Supplement: Supplementary file 2 — Additional file 2: full-length blot gels are presented in Supplementary Figure 2E. [file 12868_2022_755_MOESM2_ESM.tif]
